# Two case reports of bilateral vertebral artery tortuosity and spiral twisting in vascular vertigo

**DOI:** 10.1186/1471-2377-14-14

**Published:** 2014-01-16

**Authors:** Zhang Hong-tao, Zhang Shu-ling, Zhang Dao-pei

**Affiliations:** 1 Department of Neurology, Zhengzhou People’s Hospital, Zhengzhou 450003, China

**Keywords:** Vertebral artery, Basilar artery, Tortuosity, Vertigo

## Abstract

**Background:**

Tortuous blood vessels are commonly seen in the cerebral arteries. The association between vertebrobasilar artery tortuosity and vascular vertigo remains obscure.

**Case presentation:**

We describe two patients with vascular vertigo who had bilateral curving and spiral looping in multiple segments of the vertebral arteries and also exhibited basilar artery tortuosity. Both patients had cerebrovascular risk factors and exhibited clinical features of vertigo with high severity, slow recovery, and recurrent tendencies. Contrast enhanced magnetic resonance angiography of the neck showed bilateral tortuosity in the V2 segments and spiral twisting in the V4 segments of the vertebral arteries, and basilar artery curving. No obvious sign of atherosclerotic stenosis was found in the vertebrobasilar arteries and no abnormalities were observed in the internal carotid arteries. Transcranial Doppler ultrasound showed decreased blood flow in tortuous vertebrobasilar arteries. Brainstem auditory evoked potentials showed that the interpeak latencies (IPL) of waves III-IV were prolonged, with a ratio of IPL III-V/IPL I-III > 1.

**Conclusions:**

Vertebrobasilar tortuosity in combination with cerebrovascular risk factors may lead to vascular vertigo in these patients.

## Background

Tortuous blood vessels are commonly seen in the cerebral arteries. While mild tortuosity is not commonly associated with clinical symptoms, severe tortuosity can lead to vertigo [1]. With the advance and wider application of reliable and noninvasive imaging technologies such as magnetic resonance angiography (MRA), an increasing number of tortuous vessels are being detected. It has been reported that hemodynamic abnormalities caused by vascular tortuosity are different from those induced by atherosclerotic stenosis [2]. However, the etiology and pathogenesis of torturous vessels are poorly understood [3]. A high incidence of vascular abnormalities and tortuosity in the vertebrobasilar artery has been reported [4], and severe tortuosity can result in poor blood supply to the brain, leading to clinical symptoms of transient ischemic attack [5]. Artery tortuosity is associated with aging and hypertension, and patients with hypoplastic vertebral arteries who have cardiovascular risk factors such as hypertension and diabetes are susceptible to vascular vertigo due to hemodynamic abnormalities in the posterior circulation [6]. Here, we describe two cases of bilateral vertebral artery tortuosity with detailed clinical and imaging findings, presenting evidence for the association between vertebral artery tortuosity and vascular vertigo.

## Case presentation

### Case 1

A 79-year-old male was admitted with a three day history of paroxysmal vertigo, diplopia, nausea and weakness occurring one to two times daily, lasting three to five minutes and not associated with tinnitus, dysphagia, hearing loss, focal sensory symptoms or altered consciousness. He had been hospitalized on at least two occasions for these episodes and symptoms were prolonged lasting more than ten days. He had a history of coronary artery disease but no history of diabetes or hypertension. He was on an antiplatelet and a statin but was non-compliant and taking them irregularly. On examination, he was amnesic for recent events, had a torsional nystagmus and a positive Romberg’s sign. The remainder of his neurological examination was unremarkable. Dix-Hallpike and head impulse tests were both negative.

**Figure 1:**
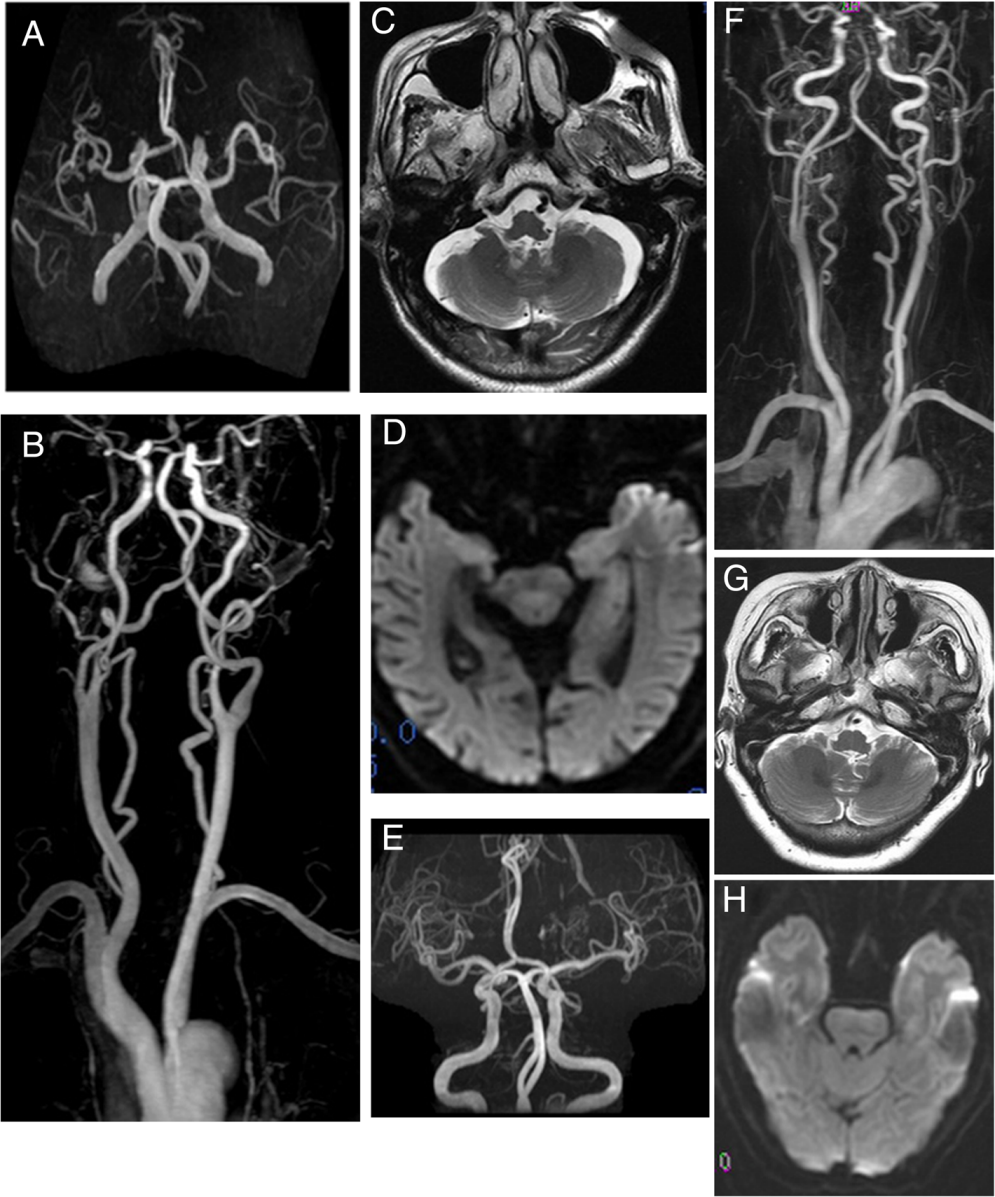
**MRA and CEMRA images of case 1 (A-D) and case 2 (E-H).** No abnormality was observed in the internal carotid arteries. **A**, **E**) Angiograms show tortuosity of the basilar artery. **B**, **F**) CEMRA images show bilateral tortuosity in the V1-V2 segment of the vertebral artery, and bilateral spiral twisting in the V4 segments of the vertebral arteries without obvious stenosis. **C**, **G**) Cross-sectional images of MRA show that the left and right vertebral arteries lie adjacent to each other. **D**, **H**) Acute brain infarction is not observed.

There was no evidence of acute infarction on MRI brain. Contrast-enhanced MRA (CEMRA) of the neck showed bilateral tortuosity in the V2 segments of the vertebral arteries, and bilateral spiral twisting in the V4 segments of the vertebral arteries without obvious atherosclerotic stenosis (Figure 1A-D). Electrocardiography (ECG) was normal, but echocardiography showed left ventricular diastolic dysfunction. No abnormality was found in the anteroposterior chest radiography. A transcranial Doppler ultrasound (TCD) showed decreased blood flow in bilateral vertebral and basilar arteries. Brainstem auditory evoked potentials (BAEP) showed that the interpeak latency (IPL) of waves III-IV was prolonged with the ratio of IPL III-V/IPL I-III > 1 (Table 1). Routine laboratory evaluation including lipid profile, glucose and glycosylated haemoglobin (HbA1C) was unremarkable. After admission, the patient was managed conservatively and treated with antiplatelets and statin. His symptoms resolved over a week.

**Table 1:** Brainstem auditory evoked potential (BAEP) results of the two patients

**BAEP**	**Peak latency**			**Interpeak latency**			**III-V/I-III**
	**(s)**			**(s)**			
	**I**	**III**	**V**	**I-III**	**III-V**	**I-V**	
Case 1	1.68	3.84	6.03	2.16	2.19	4.35	1.01
Case 2	1.67	3.85	6.06	2.18	2.21	4.39	1.01

### Case 2

A 68-year-old female was admitted with a seven day history of paroxysmal vertigo, an ataxic gait, nausea and weakness lasting approximately one minute and occurring several times daily, and not associated with tinnitus, dysphagia, hearing loss, focal sensory symptoms or altered consciousness. She was hospitalized for a recent attack six months ago and had been discharged seven days after the treatment. She had a past history of hypertension and diabetes. She was taking an oral glucose lowering medication and antihypertensive medication. On examination, she had right-beating nystagmus of both eyes, lasting for several seconds during vertigo attacks and a positive Romberg’s sign. The remainder of her neurological examination was unremarkable. Dix-Hallpike and head impulse tests were both negative.

There was no evidence of acute infarction on MRI brain. Cervical CEMRA showed bilateral looping in the V2 segments of the vertebral arteries, and bilateral spiral twisting in the V4 segments of the vertebral arteries without obvious atherosclerotic stenosis (Figure 1E-H). ECG and echocardiography were normal. No abnormality was found in the anteroposterior chest radiography. TCD showed decreased blood flow in the left and right vertebral arteries. BAEP showed that the IPL I-III and IPL III-V were prolonged with the ratio of IPL III-V/IPL I-III > 1 (Table 1). Routine laboratory evaluation including total cholesterol, and light density lipoprotein (LDL) cholesterol was unremarkable. The concentrations of blood triglycerides was 2.34 mmol/L. The fasting blood glucose concentration was 6.5 mmol/L, and the glycosylated hemoglobin level was 6.71%. After admission, the patient was managed conservatively and treated with antiplatelets, statin, antihypertensive drugs, and antihyperglycemic therapy. Her symptoms resolved over a week.

## Discussion

Vascular vertigo is frequently encountered in clinical practice but less commonly recognised [7,8]. We describe two elderly patients with vascular vertigo who had cardiovascular risk factors, including a history of hypertension and diabetes in one patient and a history of coronary disease in the other patient. The possibility of a peripheral cause for vertigo was considered less likely from Dix-Hallpike and head impulse tests. According to the diagnostic criteria for vertigo set by the Committee of Hearing and Equilibrium in the USA, the vertigo severity was high (grade 3 and grade 4) with a long recovery time (eight days and ten days) with a tendency to recur for the two patients. According to the diagnostic criteria for tortuosity in the trunk of the basilar artery with MRA [9], the basilar artery was graded 1 in one patient and 3 in the other patient. Neuroimaging of both patients was unusual, revealing spiral twisting in the V4 segment around the vertebral arteries. Cervical CEMRAs showed that tortuosity in the V1 and V2 segments of the vertebral arteries occurred in both patients including one case with looping, and the other case with curving in the V2 segment [10].

It has been reported that vascular tortuosity and hypoplasia in the vertebral or basilar arteries promote posterior circulation infarctions via altering the hemodynamics or accelerating atherosclerosis [11,12]. In addition, abnormal BAEP readings have been associated with vascular vertigo in patients with tortuous vertebrobasilar arteries [1], and vertebrobasilar artery hypoplasia has been found in a patient with vascular vertigo [13]. Abnormal BAEP readings are associated with vertebrobasilar transient ischemic attacks [1,14,15], and are therefore considered a good measure of brainstem function. The occurrence of tortuosity in the entire vertebrobasilar system with bilateral spiral twisting in the V4 segments of the vertebral arteries, as reported in these case studies, is relatively rare. The clinical features and the BAEP evidence showing ischemia in the brain stem suggest that tortuosity and spiral twisting of the vertebrobasilar arteries in conjunction with cerebrovascular risk factors contributed to the vascular vertigo in the two patients. The features of vertigo, such as high severity, slow recovery, and recurrent tendency, may be associated with curving and looping in multiple segments of the arteries, leading to a hemodynamic abnormality in the arteries that is resistant to medical treatments. Vertigo attacks can recur when the tortuous artery with compensatory regulatory dysfunction fails to meet the increased blood flow demand in the brain. If tortuosity is only present in one segment of the artery and/or no vascular risk factors exist, vascular vertigo may not occur. However, vascular vertigo is prone to occur in patients with cerebrovascular risk factors and tortuosity in multiple segments of the artery. Interventional treatments can improve blood perfusion, thereby alleviating clinical symptoms in patients with atherosclerotic stenosis [16]. However the effect of intervention on mechanical stenosis from vascular tortuosity is not well established [17].

The mechanisms resulting in vertebrobasilar artery tortuosity remain unclear [3]. It has been reported that vertebrobasilar artery tortuosity is associated with connective tissue disorders [18], reduced elasticity and degeneration of blood vessels, and vascular wall shear stress [3,19]. Vascular risk factors such as hypertension, diabetes and lipid metabolism disorders can promote atherosclerosis, aging, and degeneration of blood vessels, thereby aggravating vertebrobasilar artery tortuosity [20]. In the present study, both patients were elderly with no connective tissue disorders, but with cerebrovascular risk factors, and had tortuosity in multiple segments of the vertebrobasilar arteries with spiral looping in the V4 segments of the vertebral arteries, suggesting that reduced elasticity and degeneration of the blood vessels was the likely cause of blood vessel tortuosity in these patients. However, congenital variants of the vertebral artery are common. For example, the vertebral artery can arise from an aortic arch [21], or have abnormal duplicated branches [22]. Additionally, the left vertebral artery is dominant in 50% of the population, the right in 25%, and two vertebral arteries with similar caliber account for the remaining 25% of cases [5]. Furthermore, emerging evidence suggests that vertebral artery hypoplasia may contribute to ischemic events and is closely associated with both atherosclerotic and prothrombotic processes, especially when other risks factors are present [5]. Therefore, we cannot exclude the possibility of anatomical variants for tortuous blood vessel formation in these patients. The spiral twisting could have been a congenital variant since it is unlikely that an acquired spiral twist could occur within a closed vascular system. Moreover, the pathogenesis of blood vessel spiral twisting requires further investigation.

## Conclusion

In conclusion, we describe two elderly patients with vascular vertigo, who had vertebrobasilar tortuosity and cerebrovascular risk factors were described. The attacks of vertigo experienced by these patients most likely resulted from a combination of vertebrobasilar tortuosity and cerebrovascular risk factors. Further studies on the roles of cerebrovascular risk factors in hemodynamic abnormalities of patients with blood vessel tortuosity will highlight therapeutic targets for the prevention and treatment of cerebrovascular diseases.

### Patient consent

Written informed consent was obtained from both patients for publication of this case report and any accompanying images. A copy of the written consent is available for review by the Editor-in-Chief of this journal.

## Abbreviations

IPL: Interpeak latencies; MRA: Magnetic resonance angiography; MRI: Magnetic resonance imaging; CEMRA: Contrast-enhanced MRA; ECG: Electrocardiography; TCD: Transcranial doppler ultrasound; BAEP: Brainstem auditory evoked potentials; LDL: light density lipoprotein.

## Competing interests

Zhang Hong-tao carried out the clinical and imaging data. Zhang Shu-ling participated in the examination of BAEP and TCD. Zhang Hong-tao and Zhang Shu-ling contributed equally to ensuring the integrity of the data, participation in the study conception, design, and data analysis. Zhang Dao-pei drafted the manuscript. All authors read and approved the final manuscript.

## Authors’ contributions

The authors declare that they have no competing interest.

## Pre-publication history

The pre-publication history for this paper can be accessed here:

http://www.biomedcentral.com/1471-2377/14/14/prepub
